# Wood Polymer Composite Based on Poly-3-hydroxybutyrate-3-hydroxyvalerate (PHBV) and Wood Flour—The Process Optimization of the Products

**DOI:** 10.3390/ma17122955

**Published:** 2024-06-17

**Authors:** Wiesław Frącz, Andrzej Pacana, Dominika Siwiec, Grzegorz Janowski, Łukasz Bąk, Paulina Szawara

**Affiliations:** 1 Department of Materials Forming and Processing, Rzeszow University of Technology, Powstancow Warszawy 8, 35-959 Rzeszow, Poland; wf@prz.edu.pl (W.F.); gjan@prz.edu.pl (G.J.); lbak@prz.edu.pl (Ł.B.); p.szawara@prz.edu.pl (P.S.); 2 Department of Manufacturing Processes and Production Engineering, Rzeszow University of Technology, Powstancow Warszawy 8, 35-959 Rzeszow, Poland; app@prz.edu.pl

**Keywords:** WPC biocomposites, Taguchi method, process optimization, injection molding

## Abstract

This study involved the optimization of the molded pieces manufacturing process from a poly-3-hydroxybutyrate-co-3-hydroxyvalerate biocomposite containing 30% wood flour by mass. The amount of wood flour and preliminary processing parameters were determined on the basis of preliminary tests. The aim of the optimization was to find the configuration of important parameters of the injection process to obtain molded pieces of good quality, in terms of aesthetics, dimensions, and mechanical properties. The products tested for quality were dog bone specimens. The biocomposite was produced using a single-screw extruder, whereas molded pieces were made using an injection molding process. The Taguchi method was applied to optimize the injection molding parameters, which determine the products quality. Control factors were selected at three levels. The L27 orthogonal plan was used. For each set of input parameters from this plan, four processing tests were performed. The sample weight, shrinkage, elongation at break, tensile strength, and Young’s modulus were selected to assess the quality of the molded parts. As a result of the research, the processing parameters of the tested biocomposite were determined, enabling the production of good-quality molded pieces. No common parameter configuration was found for different optimization criteria. Further research should focus on finding a different range of technological parameters. At the same time, it was found that the range of processing parameters of the produced biocomposite, especially processing temperature, made it possible to use it in the Wood Polymer Composites segment.

## 1. Introduction

Due to the increasing ecological awareness of society resulting from the need to reduce polymer materials of petrochemical origin, biodegradable and natural polymer materials are receiving more and more attention. An example of this type of polymer is poly(3-hydroxybutyric acid-co-3-hydroxyvaleric acid) (PHBV), which is a promising alternative to standard petroleum-based plastics. It belongs to the group of PHA polymers (polyhydroxyalkanoates)—biodegradable, of natural origin, produced in the mitochondria of bacteria as a reserve material [1,2]. Compared to another polymer from this group, i.e., poly(3-hydroxybutyric acid (PHB), PHBV is characterized by reduced stiffness and brittleness, higher elongation at break and increased tensile strength. Changing the content of valerate (HV) in the polymer chain can significantly reduce melting temperature, increasing the difference between the melting temperature and the beginning of thermal decomposition, which contributes to the improvement of the processing temperature range [3,4]. In order to reduce the costs of producing the rather expensive PHBV compared to other classic materials, an interesting solution may be the development of modern biocomposites based on the above-mentioned material with fillers of plant origin. The use of this type of filler can provide many advantages to the developed biocomposites [5]. They can often improve the mechanical properties of the developed biocomposites, are also cheaper than synthetic fillers, and can contribute to reducing the consumption of fossil raw materials and greenhouse gas emissions [6]. The disadvantages of this type of materials are also worth mentioning. They have undoubtedly high hygroscopicity and a fairly large range of properties, which may be caused by the conditions of cultivation, harvesting time, processing, and storage methods [7]. In addition, other factors determining the dispersion of properties of this type of materials, i.e., natural fillers, can be indicated. Filler particles can have various sizes and shapes, from long-length and small-diameter fibers to isometric-shaped particles. These materials consist of different contents of cellulose, hemicellulose, lignin, and other substances within one species of plant filler. Moreover, they may have different surface properties, such as roughness and hydrophilicity, which affect the wettability and adhesion of the filler to the matrix [8,9]. The process of producing this type of composite and its processing also determines its properties and distribution. The mixing method used to prepare the material for the extrusion process and the mixing itself during the process are crucial for the even distribution of the filler in the polymer matrix. Improper mixing may lead to agglomeration of filler particles, which may negatively affect the properties of the biocomposite [10,11]. Temperature conditions, pressure, and processing time may affect the properties of the biocomposite. Improper processing conditions may lead to degradation of the filler or polymer matrix, which may negatively affect the properties of the biocomposite. Additives such as stabilizers, compatibilizers, and release agents can be used to improve the properties of the biocomposite. An improper selection or a certain amount of additives may negatively affect the properties of the biocomposite [12,13]. The variability in the properties of biocomposites with fillers of plant origin creates a number of challenges for producers and users of these materials. The variability of the properties of plant-derived fillers may make it difficult to standardize the biocomposites production process and may limit their use in some areas. Moreover, it is necessary to use control tests at every stage of biocomposite production to ensure their compliance with specific specifications [14].

The course of the plastic injection molding process affects the behavior of the material during flow and its solidification process. The forming process may also result in product defects such as deformation, shrinkage, welding lines, and traces of overheating. Among the defects mentioned, deformation and shrinkage have the greatest impact on the quality of the product [15,16,17,18].

There are two ways to reduce post-injection deformation and shrinkage in the injection molding process. In the first method, the product and mold are appropriately designed to reduce deformation and shrinkage values. In the second method, the process parameters were changed to obtain minimal deformation and shrinkage [19,20,21].

Product quality is an important issue that is constantly being addressed [22,23]. Since post-injection deformations and shrinkage are the two main causes that affect product quality, literature analysis was performed from this point of view. Mirigul et al. investigated the possibilities of influencing the shrinkage of injection moldings using the Taguchi, ANOVA, and neural network methods [24]. This study utilized a rectangular sample with dimensions of 110 × 10 × 3.2 mm for experiments and used two types of materials, polypropylene (PP) and polystyrene (PS). These materials were injected into the sample mold cavity under various process parameters: injection temperature, injection pressure, holding pressure, and holding time. Based on the analysis results, an optimal set of parameters that cause minimal sample shrinkage was determined. The optimal set of parameters included a melting temperature of 260 °C, injection pressure of 60 MPa, pressing pressure of 50 MPa, and pressing time of 15 s, which gave a minimum shrinkage of 0.937% for PP and 1.224% for PS. It was found that the most significant parameters were the holding pressure and melt temperature, respectively. The injection pressure had the least influence on the shrinkage value. Tuncay et al. presented the minimization of the strain index in thermoplastic injection mold parts using the Taguchi optimization method [25]. The aim of this study was to minimize the deformation index (expressed as the ratio of the secondary to primary deformation modulus) in terms of process parameters of plastic products with different types of rib cross-sections and rib angles using the Taguchi optimization method. Based on process parameters such as injection temperature and holding pressure, in addition to the types of rib cross-sections, a series of analyzes were performed using the deformation data. The polymer materials chosen were PC/ABS, polyoxymethylene (POM), and polyamide (PA66). The Taguchi optimization method was used to analyze the injection process, taking into account three-level factorial planning. Taguchi orthogonal systems, the signal-to-noise ratio (S/N), analysis of variance (ANOVA) were used to find the optimal levels of influence of process parameters on the strain index for the analyzed product. An analysis test was performed with optimal levels of process parameters to obtain the validity of the Taguchi method. From this, it can be concluded that the Taguchi method is suitable to solve the quality problem encountered in injection-molded products. Subramanian N.R. presented the optimization of deformations for an optical housing using the same method [26]. Babur O. and Ibrahim S. studied the deformation and analyzed the structure of molded pieces [27]. Tuncay E. studied the application of the Taguchi optimization technique in determining the parameters of the plastic injection molding process for a thin-walled product [25]. The analysis of the results performed in this work shows that deformations and shrinkage decreased by 2.17% and 0.7%, respectively. A verification test was also performed to prove the effectiveness of the Taguchi method after determining the optimal thin-film plastics for plastic injection molding. In the study by Hasan O. [28], optimization performed using the Taguchi method turned out to be sufficient to solve the problem of deformation associated with the shrinkage of thin-layer elements made of polymer materials. Eghbal H. and Abu B.S. [29], using the same method, analyzed the deformation and shrinkage properties of injection-molded polymer composite micro-gears using numerical simulation aided by the Taguchi method. Azaman M.D. [30] and other researchers investigated post-injection shrinkage and deformation in the processing of wood-filled, thin-walled polypropylene composite parts produced by injection molding. In this study, injection molding of shallow, thin-walled products (0.7 mm thick) composed of polypropylene PP + 50% wood composites was simulated. Volume shrinkage and deformations in thin-walled elements were evaluated for various process conditions. Mold temperature, cooling time, pressure, and pressing time were taken into account. The analysis showed that cooling time and pressing time had less impact on shrinkage and deformation; nevertheless, their optimal levels for both parameters were required in the forming process for a thin-walled product in order to obtain good quality. The volumetric shrinkage was lower near the injection point than at the end of the flow path. The test results also showed that volumetric shrinkage correlated with the deformation measured on the molded part. Optimal ranges of processing parameters (40–45) °C for mold temperature, 20–30 s for cooling time and 15–20 s for pressing time, were determined to obtain the best results while maintaining the lowest possible volume shrinkage and deformations. Moreover, Sanchez R., Aisa J., and Martinez A. [31] studied the relationship between the cooling system and deformation in injection molding. This study focused on the impact of the cooling effect on product deformation using non-contact techniques in several areas. The results allow one to visualize changes in warpage when injection temperature, cooling time, or cooling conditions are different. Babur O. studied the optimization of injection parameters on the mechanical properties of a sample with a joint line for polypropylene using the Taguchi method [32]. Kamber S.O. and co-authors presented the influence of pressure in the mold cavity and its temperature on the quality of final products [33]. In these tests, the pressure and temperature of the mold surface were measured and recorded using pressure and temperature sensors by means of a Kistler CoMo 2869A device. The impact of the analyzed factors on the product quality was experimentally tested. The results of this experimental study indicate that mold pressure and temperature are the dominant factors determining the quality of the injection molded product. Kuo M.T. studied the influence of process parameters on the surface quality of optical lenses [34]. Similarly, using the same method, Hamdy H. examined the effect of a cooling system on the shrinkage and temperature of the polymer through injection molding [35]. The Taguchi method is one of the optimization methods [36]. The recognized use of this method in the optimization of plastic processing processes makes it possible to optimize the injection process in order to obtain shapes for testing the properties of new materials—in this case, WPC biocomposites. The Taguchi method has been successfully used in industry and has completely changed the perspective on quality improvement activities [37]. It includes the design and testing of the system based on the materials selected by the engineer and the nominal parameters of a process or a product [38]. The Taguchi method is used when there is an intermediate number of variables (from 3 to 50), few interactions between variables, and when only a few variables are significant [39,40,41].

The Taguchi method has been used in the plastics industry for years. The aim of this study was to use it to optimize the process of molded pieces producing from the proposed new composite and to present the methodology for selecting process parameters, which mostly affect the quality of the produced products. The aim of the optimization carried out for many criteria was to find out if it is possible to obtain a configuration of processing parameters for which the quality of the products in every aspect of the material’s mechanical properties, shape, and dimensional accuracy will be the same.

## 2. Materials and Methods

### 2.1. Research Materials

In the research, a Wood Polymer Composite was produced from poly-3-hydroxybutyrate-co-3-hydroxyvalerate (PHBV) and wood flour.

Wood flour produced by Rettenmaier Poland Ltd–JRS (Warsaw, Poland) with particle sizes of 70–150 µm was applied to improve the mechanical properties of PHBV. Wood filler is often applied in the form of fibers in the production of polymer composites [42,43,44,45].

The ENMAT Y1000 biopolymer of Helian Polymers (Belfeld, The Netherlands) in powder form was selected for testing [46]. The properties of the biopolymer applied are presented in Table 1. The ENMAT Y1000 was used in powder form.

### 2.2. Production of Composite and Test Samples

**Figure 1:**
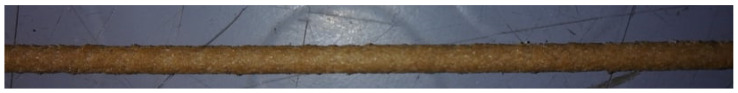
PHBV composite extrudate—wood flour with a circular cross-section, obtained using an EHP 25E single-screw extruder.

In the tests, a WPC composite with a mass fraction of wood flour of 30% was produced. The composite was extruded on an EHP 25D single-screw extruder from ZAMAK-MERCATOR company (Skawina, Poland). The extrudate (Figure 1) was made, using a G16/32-II granulator from ZAMAK-MERCATOR company (Skawina, Poland), into a cylindrical granulate, approx. 2 mm in diameter and approx. 3 mm long.

The composite was produced using only one temperature profile of the extruder zones, presented in Table 2.

The extruder charging temperature was maintained at 50–55 °C. A constant temperature profile was used (zones 4–5), which was determined using a narrow processing window of the prepared composite. Lowering the temperature results in excessive load on the extruder drive (over 95% of the nominal load), while increasing the temperature significantly reduces the viscosity of the composite in head, which creates problems in the granulate formation process (cooling and removal of the extrudate). It was assumed that the composite could be extruded for a drive system load lower than 95% of the extruder’s nominal load. The process was carried out at screw speeds of approximately 60 rpm. After stabilizing the process, the instantaneous load of the drive system changed only within a few percent (plus or minus), but below 100%. The drive disconnection threshold due to overload of the extruder system was 110% of the nominal load.

The specimens for testing the mechanical properties were manufactured using a Dr Boy 55E injection molding machine (produced by BOY Maschines Inc., Exton, PA, USA) for a range of input parameters determined on the basis of preliminary tests. Based on the preliminary tests, it was found that some mechanical properties of the manufactured samples changed together with some changes of processing parameters. Preliminary research allowed us to select sets of process parameters and the content of wood flour that produced specimens with a good shape and dimension accuracy, and the injected composite showed good processing and mechanical properties. Based on observations and strength tests of the obtained specimens, the ranges of important injection parameters were determined. The factors used to control the process (processing parameters) and their levels of variability adopted for research are listed in Table 3.

In order to minimize the number of experiments performed, the Taguchi method was used. Minitab ver.14 software was applied to generate the orthogonal plan. Based on Table 3, an L27 orthogonal plan was generated containing 27 configurations of control factors. This plan is presented in Table 4.

For each set of processing parameters from the L27 orthogonal plan, four technological tests were performed under production conditions. The samples for mechanical property tests were made in accordance with the ISO 527-1 standard [47]. Taking into account that the mold used was a two-cavity, a total of over 200 samples were produced in four series. The results were analyzed for cycles 3 and 4 of each parameter configuration.

## 3. Results and Discussion

### 3.1. Accuracy Testing of Molded Parts

**Figure 2:**
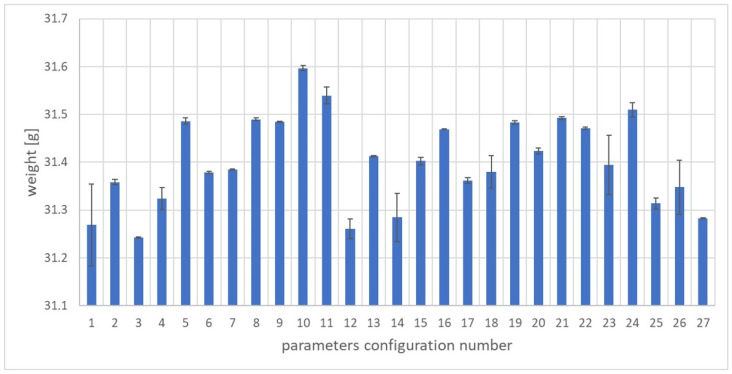
Arithmetic mean and standard deviation of sample weight for individual sets of adjustable parameters.

Preliminary tests concerned the weight of the samples. Deviations from the optimal mass indicate an increase or a decrease in the dimensions of the molded part, i.e., their dimensional inaccuracy. Such tests are most often carried out in industry due to quick measurement. The weight and length of the samples were measured 24 h after they were made. The samples were weighed on a AS.110 PLUS laboratory balance by RADWAG company (Radom, Poland) with an accuracy of 0.0001 g. The arithmetic mean and standard deviation of sample weight for each set of parameters are shown in Figure 2. The lowest sample weight was obtained for the third set of adjustable parameters and amounted to 31.24 g. The highest mass value was 31.59 g for the 10th set of adjustable parameters. The average mass value of all injections performed was 31.40 g and the median was 31.39 g. The highest value of the standard deviation was obtained for the first set of adjustable parameters and was 0.085 g. In turn, the lowest value of the standard deviation was recorded for the seventh set of adjustable parameters and was 0.00075 g. The obtained results indicate a high uniformity of the product mass for variable adjustable parameters. The difference between the largest and smallest mass values for the discussed configurations is only 0.34, which is approximately 1.12% of the average product mass.

Then, based on measurements of the dimensions of the molded part and the cavity, volumetric shrinkage was calculated. The shrinkage measurement was performed 24 h after the samples were produced. In order to determine the dimensions of the forming cavity at the temperatures used in individual configurations, the following relationship was applied:

(1) $$L_{F}=L_{F}\left[1+\alpha \left(T_{F}-T_{0}\right)\right]$$

where *L_F_*—cavity dimension at temperature *T*_0_, *α*—thermal expansion coefficient, *T_F_*—mold temperature, and *T*_0_—ambient temperature.

The value of volume shrinkage was calculated from the following relationship:

(2) $$S_{v}=1-(1-S_{II})(1-S_{⊥})(1-S_{T})$$

where *S_II_*—parallel shrinkage, *S*_⟂_—perpendicular shrinkage, and *S_T_*—thickness shrinkage of the molded part.

**Figure 3:**
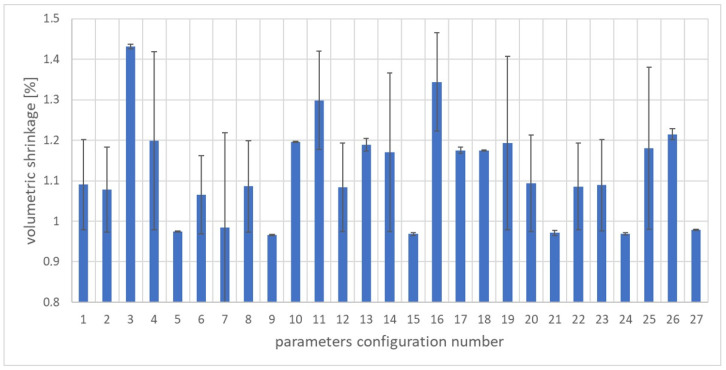
Arithmetic mean of volumetric shrinkage and standard deviation for individual parameter sets (1–27) according to the orthogonal table L27.

The arithmetic mean of four production cycles and the standard deviation for volume shrinkage for subsequent sets of parameters are presented in Figure 3.

The lowest value of the sample volume shrinkage was obtained for the ninth set of adjustable parameters and amounted to 0.96%. In turn, the highest shrinkage value was 1.43% for the third set of adjustable parameters. The average shrinkage value for all injections was 1.12% and the median was 1.09%. The highest standard deviation value was obtained for the seventh set of adjustable parameters and amounted to 0.23%. In turn, the lowest standard deviation value was recorded for the 10th set of adjustable parameters and amounted to 0.0012%. The obtained results indicate a large spread and variability of product shrinkage for variable adjustable parameters. The difference between the largest and smallest shrinkage values for the discussed configurations was as much as 0.046% of the volume shrinkage value, which is approximately a 41.47% change compared to the calculated average product shrinkage.

### 3.2. Mechanical Properties Tests

**Figure 4:**
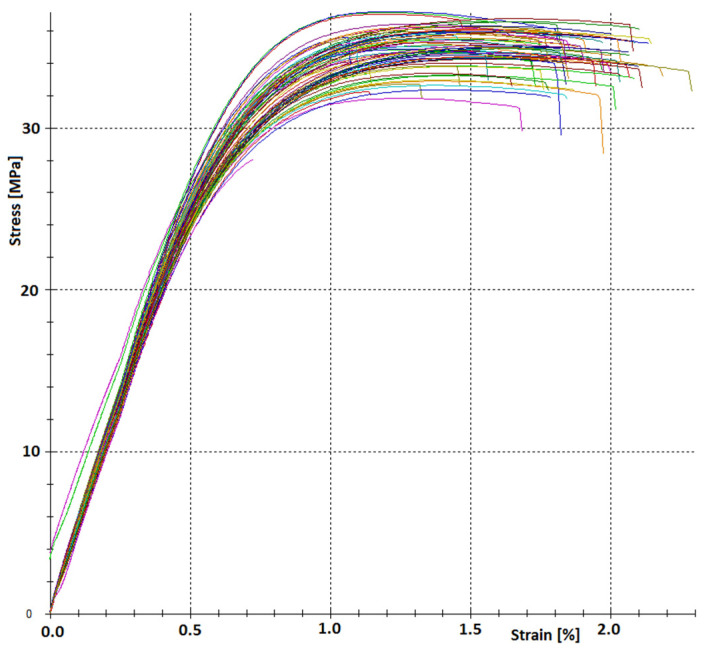
Examples of tensile curves for the PHBV–wood flour composite (30% by mass) made with various processing parameters (Table 4)—graph directly from the testing machine.

Strength tests confirmed the influence of process parameters on the mechanical properties of manufactured products (specimens). The strength test chart is presented in Figure 4. Due to the large amount of data, no key is provided in this figure. The significant discrepancy between the tensile curves obtained for different samples confirms this effect on the mechanical properties of the injected composite.

**Figure 5:**
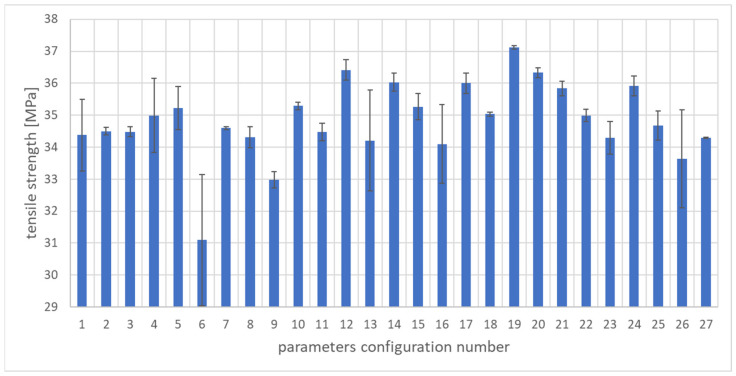
Arithmetic mean of tensile strength and standard deviation of samples for each set of parameters.

**Figure 6:**
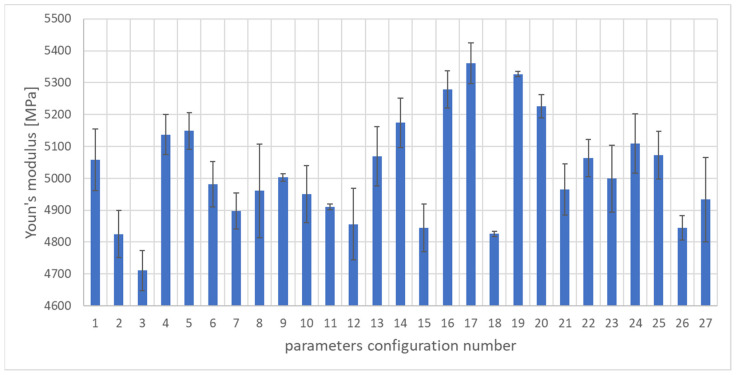
The arithmetic mean of the Young’s modulus along with the standard deviation of samples for each set of parameters.

**Figure 7:**
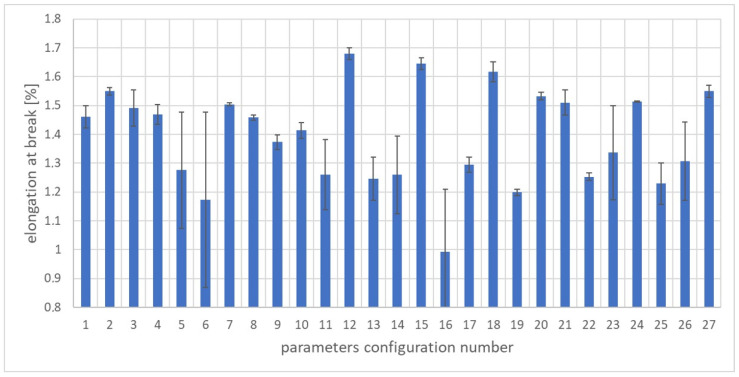
The arithmetic mean of the sample elongation along with the standard deviation for each set of parameters.

To assess the quality of the WPC composite, the mass of the molded part and the following mechanical properties of the biocomposite were selected: elongation at break, tensile strength, and Young’s modulus. Strength tests were performed on a Zwick/Roell Z100 testing machine ( produced by Zwick Roell, Ulm, Germany) in accordance with the ISO 527-1 standard. Figure 5, Figure 6 and Figure 7 show the properties of the molded pieces obtained for various configurations of adjustable parameters as arithmetic averages for four production cycles, produced after the parameters in the injection molding machine system had been stabilized.

The lowest value of tensile strength was obtained for the sixth set of adjustable parameters and amounted to 30.46 MPa. In turn, the highest value was 37.17 MPa for the 19th set of adjustable parameters. The average value of all injections performed was 34.80 MPa and the median was 34.96 MPa. The highest standard deviation value was obtained for the sixth set of adjustable parameters and amounted to 2.44 MPa. In turn, the lowest standard deviation value was recorded for the second set of adjustable parameters and amounted to 0.009 MPa. The results indicate a significant spread and variability of tensile strength for variable adjustable parameters. The difference between the highest and lowest tensile strength values for the discussed configurations was as much as 6.71 MPa, which is approximately a 19.29% change compared to the calculated average tensile strength value.

The values of the Young’s modulus are presented in Figure 6. The lowest value of the Young’s modulus was obtained for the third set of adjustable parameters and amounted to 4710.8 MPa. In turn, the highest value was 5361 MPa for the 17th set of adjustable parameters. The average value of all injections performed was 5019.6 MPa, and the median was 4999.3 MPa. The highest standard deviation value was obtained for the eighth set of adjustable parameters and amounted to 146 MPa. In turn, the lowest standard deviation value was recorded for the 19th set of adjustable parameters and amounted to 7.92 MPa. The results indicate a very small range and average variability of tensile strength for variable setting parameters. The difference between the highest and lowest tensile strength values for the discussed configurations was as much as 6.71 MPa, which is approximately a 19.28% change compared to the calculated average tensile strength value.

The amount of elongation at break of the samples was also analyzed. The results are presented in Figure 7. The lowest elongation value was obtained for the 16th set of adjustable parameters and amounted to 0.99%. In turn, the highest value was 1.68% for the 12th set of adjustable parameters. The average value of all injection cycles performed was 1.39 MPa and the median was 1.41 MPa. The highest standard deviation value was obtained for the sixth set of adjustable parameters and amounted to 0.30%. In turn, the lowest standard deviation value was recorded for the 24th set of adjustable parameters and amounted to 0.0022%. The results indicate a very large spread and variability of the elongation and stretching for variable adjustable parameters. The difference between the highest and the lowest elongation values for the discussed configurations was as much as 0.69%, which is approximately a 49% change compared to the calculated average tensile strength value.

### 3.3. Optimization Using the Taguchi Method

After entering the input data into Minitab, a criterion that described the type of problem being analyzed was selected. For this purpose, the Taguchi method uses the so-called signal-to-noise ratio (S/N). This parameter takes into account both the average value of the measured signal and its standard deviation.

The method of calculating S/N depends on the quality criterion being tested:

- When optimizing the shrinkage of molded parts, the primary and secondary shrinkage values should be as low as possible, so the criterion chosen was “the smaller the better”. The quality characteristics and the signal-to-noise ratio (S/N) were calculated from the following equation:

(3) $$S/N=-10\cdot \log\left(\frac{1}{n}\overset{n}{\underset{i-1}{\sum }}y_{i}^{2}\right)$$

where y_i_ is primary or secondary shrinkage.

- In the case of mass and mechanical properties, the values of the Young’s modulus, tensile strength, and relative elongation at maximum tensile stress should be as high as possible, so the criterion “the bigger the better” was chosen. The quality characteristics and the signal-to-noise ratio (S/N) were calculated from the following equation:

(4) $$S/N=-10\cdot \log\left(\frac{1}{n}\overset{n}{\underset{i-1}{\sum }}\frac{1}{y_{i}^{2}}\right)$$

where y_i_ is the Young’s modulus or tensile strength or elongation at maximum tensile stress.

In order to obtain good quality moldings from the tested PHBV–wood flour biocomposite, several criteria were taken into account. Some of them should reach the maximum value, while others should be minimized due to the use of an appropriate configuration of the adjustable parameters of the molding production process. The following optimization criteria were used:

- Weight of samples (Figure 2): optimization objective function “larger is better” selected;
- Volume shrinkage (Figure 3): optimization objective function “smaller is better” selected;
- Tensile strength (Figure 4): optimization objective function “larger is better” selected;
- Young’s modulus (Figure 5): optimization objective function “larger is better” selected;
- Elongation at break (Figure 6): optimization objective function “larger is better” selected.

The most satisfactory of the pre-selected manufacturing parameters was determined using the Taguchi optimization method. All calculations were performed in Minitab, ver. 14. First, the elongation at break value was analyzed. This property should reach its maximum value.

**Figure 8:**
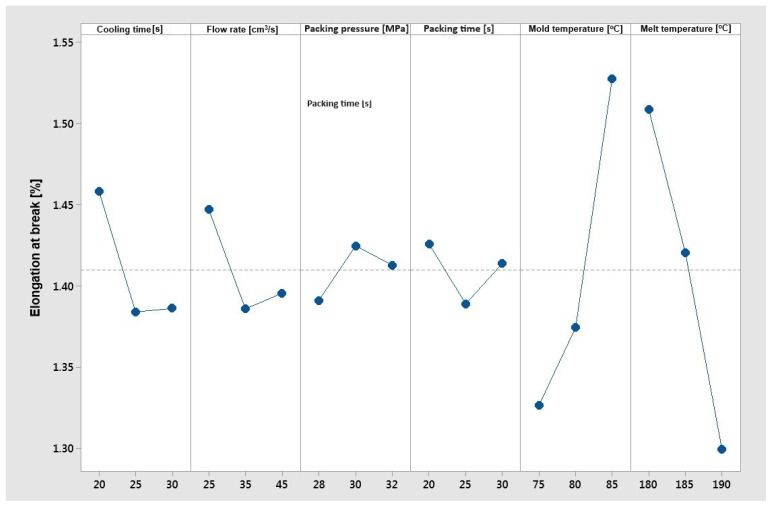
The Taguchi analysis for the criterion ‘maximum elongation at break’.

**Figure 9:**
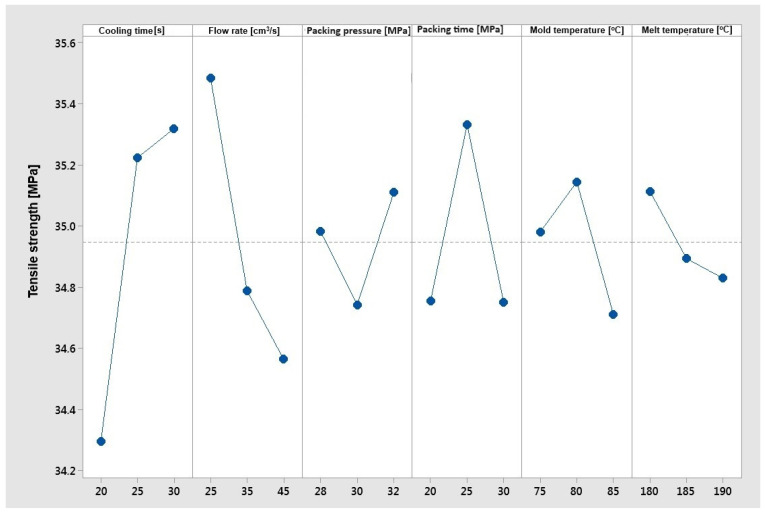
The Taguchi analysis for the criterion ‘maximum tensile strength’.

Based on Figure 8, a clear influence of changes in mold temperature and material temperature on the elongation at break can be stated. As the mold temperature increases, the elongation at break of the samples grows, and as the material temperature increases, this elongation decreases. The mold temperature has a strong influence on the filling and packing phase of the injection process. As the temperature of mold increases, the flow resistance decreases and the packing efficiency improves. Changes in mold temperature could affect the final material structure as well. Increasing the melt temperature negatively affects the elongation because of the acceleration of PHBV degradation processes. PHBV-based composites are characterized by a very small processing window; thus, the melt temperature and residence time have a high impact on mechanical properties. The negative of the melt temperature increase could also be observed for the tensile strength (Figure 9). The influence of other parameters (i.e., cooling time, flow rate, packing pressure, and packing time) is not significant and no clear impact on elongation has been demonstrated. In the case of the “larger is better” optimization objective function for elongation at break, the optimal parameters were set as follows:

– Cooling time—20 [s];
– Flow rate—25 [cm^3^/s];
– Packing pressure—30 [MPa];
– Packing time—20 [s];
– Mold temperature—85 [°C];
– Melt temperature—180 [°C].

The configuration of such parameters was not included in the established orthogonal plan (Table 4).

Based on Figure 9, it is possible to state a visible and clear influence of the trends in changes in cooling time, flow rate, and material temperature on the tensile strength. As the cooling time increases, the tensile strength grows, and as the flow rate and material temperature increase, the tensile strength decreases. The highest impact is observed for two parameters—cooling time and flow rate. The cooling time can strongly impact the material microstructure due to the cooling rate. As long as the material remains in the mold, a high cooling rate is provided. After ejection, the cooling rate strongly decrease. The negative effect of the flow rate increase on the tensile strength can be strongly related to the mechanical and thermal degradation processes of composite. The packing time, packing pressure, and mold temperature do not have a clear effect on the strength of the composite. In the case of the “larger is better” optimization objective function for tensile strength, the optimal parameters were as follows:

– Cooling time 30 [s];
– Flow rate 25 [cm^3^/s];
– Packing pressure 32 [MPa];
– Packing time 25 [s];
– Mold temperature 80 [°C];
– Melt temperature 180 [°C].

**Figure 10:**
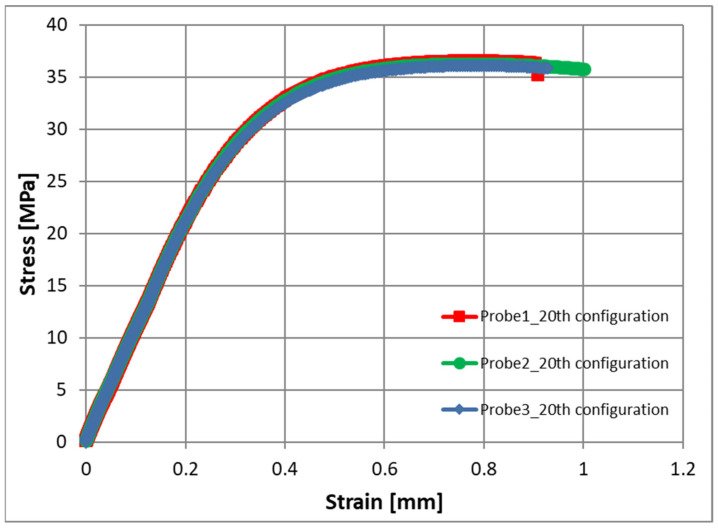
Stress–strain curves for the injected samples for the 20th configuration of adjustable parameters.

These parameters are located in a fixed orthogonal plan—this is configuration number 20 (Table 4). For configuration 20, the average tensile strength value was 36.3 MPa and the standard deviation was 0.23, which indicates a very small scatter of results. Example stress–strain curves for three samples injected for configuration 20 are shown in Figure 10. Good repeatability of mechanical properties in this case is also visible, which may indicate good, uniform filler–matrix homogenization.

**Figure 11:**
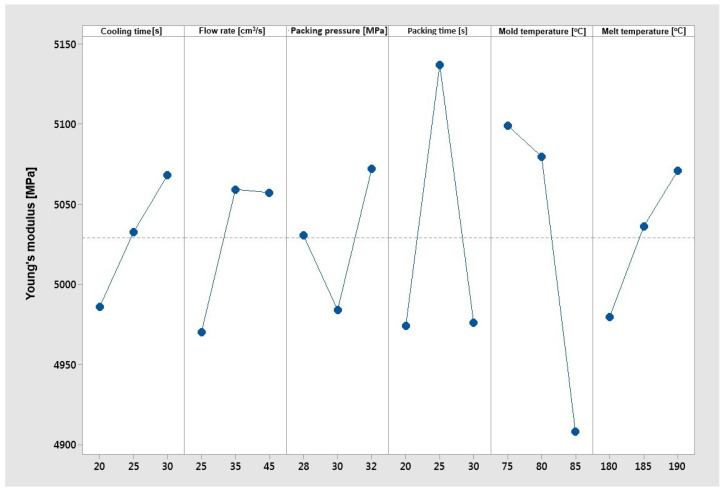
The Taguchi analysis for the criterion ‘the maximum Young’s modulus’.

Figure 11 shows a clear upward trend for changes in cooling time and material temperature, and a downward trend for changes in mold temperature. As the cooling time and temperature of the material increases, the Young’s modulus grows, and as the mold temperature increases, the Young’s modulus decreases. The relations between cooling parameters and the Young’s modulus are interesting according to properties of material without filler, where higher mold temperatures and low cooling rates promote the formation of crystallites and higher values of the Young’s modulus can be obtained.

The influence of flow rate, packing pressure, and packing time on the Young’s modulus is not clear. In order to obtain the optimal stiffness of the tested biocomposite using the “larger is better” optimization objective function, the best parameters are as follows:

– Cooling time 30 [s];
– Flow rate 35 [cm^3^/s];
– Packing pressure 32 [MPa];
– Packing time 25 [s];
– Mold temperature 75 [°C];
– Melt temperature 190 [°C].

**Figure 12:**
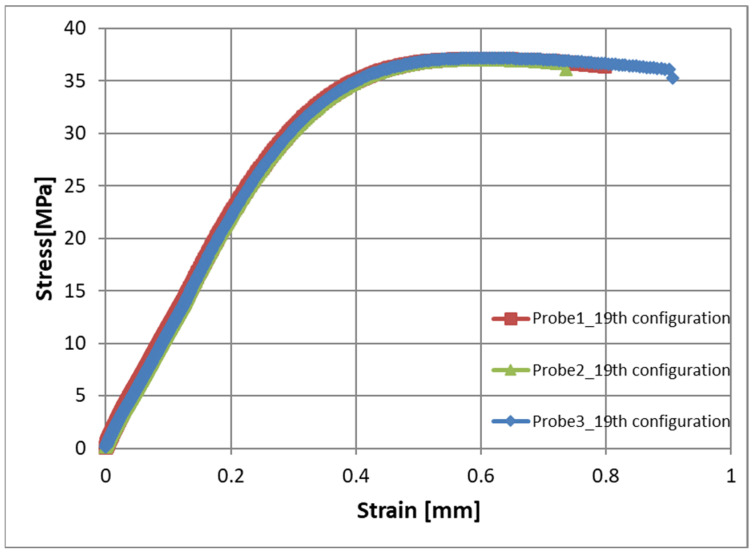
Stress–strain curves for the injected samples for the 19th configuration of adjustable parameters.

These parameters were included in the established orthogonal plan—this is configuration number 19 (Table 4). For the 19th configuration, the average Young’s modulus value was 5327 MPa, while the standard deviation was 11.3, which indicates a very small scatter of results. Example stress–strain curves for three samples injected for the 19th configuration are shown in Figure 12. Good repeatability of mechanical properties in this case is also visible, which may also indicate good, uniform filler–matrix homogenization.

**Figure 13:**
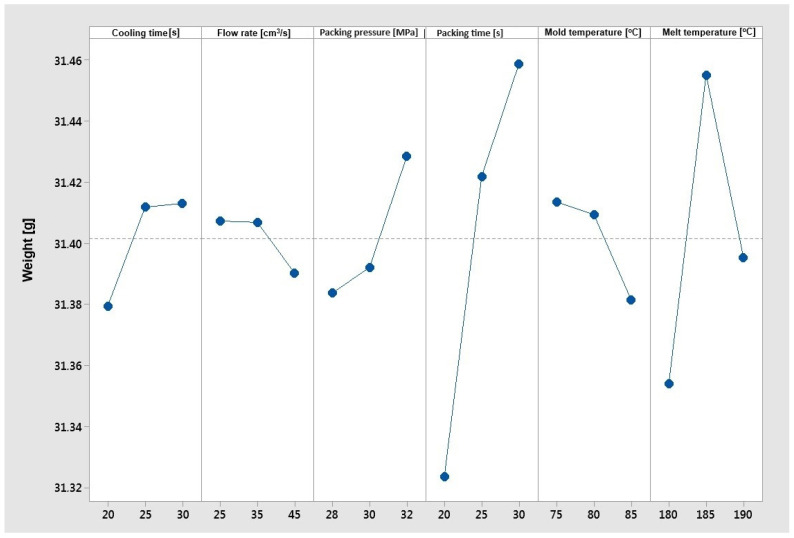
The Taguchi analysis for the criterion ‘maximum sample weight’.

Figure 13 shows a clear upward trend for cooling time, packing pressure, and packing time, and a clear downward trend for the flow rate and the mold temperature. As the cooling time and packing pressure increase, the mass of the samples grows, and as the flow rate and mold temperature increase, the mass decreases. The highest impact on weight can be observed for the packing time. The influence of the cooling time, flow rate, packing pressure and mold temperature parameters is not significant. In the case of the “larger is better” optimization objective function for the mass of samples, the best parameters are their configuration:

– Cooling time 30 [s];
– Flow rate 25 [cm^3^/s];
– Packing pressure 32 [MPa];
– Packing time 30 [s];
– Mold temperature 75 [°C];
– Melt temperature 185 [°C].

The configuration of these parameters was not found in the established orthogonal plan (Table 4).

**Figure 14:**
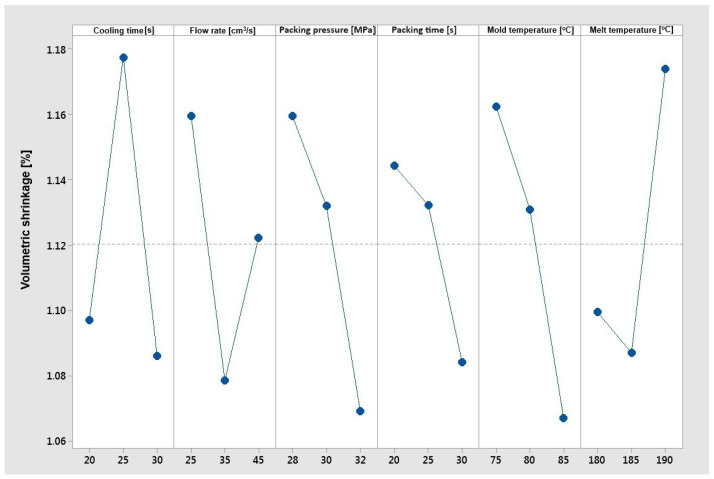
The Taguchi analysis for the criterion ‘minimum volumetric shrinkage’.

Figure 14 shows a clear downward trend for packing pressure, packing time, and mold temperature. As the pressure, packing time, and mold temperature increase, the volume shrinkage decreases. As is commonly known, the dependence between shrinkage and packing parameters is strong. The effect of the cooling rate and flow rate on shrinkage is significant and can be a cause of experiment errors. In the case of the “smaller is better” optimization objective function for volume shrinkage, the optimal parameters are as follows:

– Cooling time 30 [s];
– Flow rate 35 [cm^3^/s];
– Packing pressure 32 [MPa];
– Packing time 30 [s];
– Mold temperature 85 [°C];
– Melt temperature 185 [°C].

These parameters are very close to the assumed parameters in the orthogonal plan (configuration No. 24—Table 4). The difference only concerns the value of the clamping pressure. It should have a value of 32 MPa. For the 24th configuration, the average volumetric shrinkage value was 0.98%, while the standard deviation was 0.003, which indicates a very small scatter of results.

## 4. Summary and Conclusions

This study presents the optimization of the molded pieces manufacturing process based on the Taguchi method. In this research, a PHBV composite with a 30% wt. of wood flour was used. The Taguchi optimization method allowed us to reduce the number of experiments in determining the optimal processing parameters. The adopted range of parameters from the orthogonal table was determined on the basis of preliminary observations of the injection molding process stability.

Five optimization criteria were used to find the set of optimal processing parameters. For three optimization criteria, i.e., tensile strength, the Young’s modulus, and shrinkage, the optimal configurations of the applied parameters are represented in the given configurations applied in the L27 orthogonal table. This table effectively reduces the number of attempts. In the case of the other two criteria, i.e., sample weight and elongation at break, the configuration of optimal parameters was not represented in table L27. Tests performed in accordance with these parameters confirmed the correctness of the calculated processing parameters.

No configuration of processing parameters was found that would ensure that all criteria were met. Instead, this method provides an answer as to which of the adopted control factors (process parameters) are more important and which are less important for a given criterion. The adopted criteria is sometimes mutually exclusive, e.g., tensile strength and elongation at break. Therefore, it is not always about them being fulfilled at the same time. However, it is suggested that to obtain good quality products with a thickness of 4 mm, made of the PHBV + 30 wood powder biocomposite, the following parameters should be initially used: melt temperature of 180 °C, mold temperature of 80 °C, packing time of 25 s, packing pressure of 32 MPa, flow rate of 25 cm^3^/s, and cooling time of 30 s.

This study presents the influence of injection parameters on the mechanical properties and, thus, on the quality of injection moldings. This influence is visible, but the parameters do not always allow for the improvement of the tested properties. It should be remembered that the processed material was a composite with a natural filler, the behavior of which may be different than that of pure polymer. Processing parameters determine the packing of the filler, and its orientation, as well as the structure of the polymer, the degree of crystallinity, and the size of the crystalline forms.

Optimization of the production process of biocomposites based on the PHBV matrix with the addition of wood flour as a filler is a key aspect to obtain products with the desired properties. The essence of optimization lies in adjusting the process parameters in such a way as to achieve an optimal combination of mechanical strength and dimensional stability. By precisely controlling these parameters, it is possible to achieve even dispersion of wood flour in the PHBV matrix, which translates into improved mechanical and aesthetic properties of the final product. Additionally, process optimization can lead to reduced production costs and have a positive impact on the energy efficiency of the entire biocomposite production process. As a result, striving to optimize the production process of PHBV-based biocomposites with wood flour is a key step towards promoting sustainable development and the use of green materials in the industry.

This study highlights the possibility of producing WPC-type composites based on PHBV and wood flour. This expands the range of material diversity of composites such as Wood Polymer Composites. It is mainly the temperature range of PHBV processing that makes it possible to produce WPC composites with the addition of a wood powder filler. The PHBV processing temperature does not cause thermal degradation of wood components, which can overheat during polymer processing. At a temperature of about 200 °C, hemicellulose is degraded, which prevents the use of many types of polymer materials in the production of WPC composites. Additionally, the produced composite is biodegradable, which makes it possible to produce biodegradable products with sufficiently large operational capabilities. The analysis performed allows one to determine the range of technological parameters in creating the required product properties for various needs (material stiffness, elongation at break, shape, and dimensional accuracy of products).

## Figures and Tables

**Table 1:** Properties of PHBV in the Enmat Y1000 grade [46].

Properties	Value
Density (g/cm^3^)	1.25
Plastic stress (MPa)	31–36
Tensile strength (MPa)	39
Elongation at break (%)	2
Young’s modulus (MPa)	2800–3500

**Table 2:** Temperature of extruder zones by manufacturing the PHBV + wood flour of 30% wt composite.

T_Feed/Throat_ [°C]	T_1_ [°C]	T_2_ [°C]	T_3_ [°C]	T_Head_ [°C]
55	145	165	175	175

**Table 3:** Levels of control factor variability.

Control Factor	Level 1	Level 2	Level 3
Cooling time [s]	20	25	30
Mold temperature [°C]	75	80	85
Melt temperature [°C]	180	185	190
Packing time [s]	20	25	30
Packing pressure [MPa]	28	30	32
Flow rate [cm^3^/s]	25	35	45

**Table 4:** Adjustable processing parameters for injection molding of the WPC biocomposite.

Parameter Configuration Number	Cooling Time [s]	Flow Rate [cm^3^/s]	Packing Pressure [MPa]	Packing Time [s]	Mold Temperature [°C]	Melt Temperature [°C]
1	20	25	28	20	75	180
2	20	25	28	20	80	185
3	20	25	28	20	85	190
4	20	35	30	25	75	180
5	20	35	30	25	80	185
6	20	35	30	25	85	190
7	20	45	32	30	75	180
8	20	45	32	30	80	185
9	20	45	32	30	85	190
10	25	25	30	30	75	185
11	25	25	30	30	80	190
12	25	25	30	30	85	180
13	25	35	32	20	75	185
14	25	35	32	20	80	190
15	25	35	32	20	85	180
16	25	45	28	25	75	185
17	25	45	28	25	80	190
18	25	45	28	25	85	180
19	30	25	32	25	75	190
20	30	25	32	25	80	180
21	30	25	32	25	85	185
22	30	35	28	30	75	190
23	30	35	28	30	80	180
24	30	35	28	30	85	185
25	30	45	30	20	75	190
26	30	45	30	20	80	180
27	30	45	30	20	85	185

## Data Availability

The data presented in this study are available on request from the corresponding author.
